# CRISPR-cas technology: A key approach for SARS-CoV-2 detection

**DOI:** 10.3389/fbioe.2023.1158672

**Published:** 2023-04-12

**Authors:** Lijuan Fang, Lusen Yang, Mingyue Han, Huimei Xu, Wenshuai Ding, Xuejun Dong

**Affiliations:** ^1^ Department of Laboratory Medicine, Hangzhou Ninth People’s Hospital, Hangzhou, Zhejiang Province, China; ^2^ Medical Laboratory, Zhejiang University Shaoxing Hospital, Shaoxing, China

**Keywords:** CRISPR-Cas, nucleic acid detection, SARS-CoV-2, application, COVID-19

## Abstract

The CRISPR (Clustered Regularly Spaced Short Palindromic Repeats) system was first discovered in prokaryotes as a unique immune mechanism to clear foreign nucleic acids. It has been rapidly and extensively used in basic and applied research owing to its strong ability of gene editing, regulation and detection in eukaryotes. Hererin in this article, we reviewed the biology, mechanisms and relevance of CRISPR-Cas technology and its applications in severe acute respiratory syndrome coronavirus 2 (SARS-CoV-2) diagnosis. CRISPR-Cas nucleic acid detection tools include CRISPR-Cas9, CRISPR-Cas12, CRISPR-Cas13, CRISPR-Cas14, CRISPR nucleic acid amplification detection technology, and CRISPR colorimetric readout detection system. The above CRISPR technologies have been applied to the nucleic acid detection, including SARS-CoV-2 detection. Common nucleic acid detection based on CRISPR derivation technology include SHERLOCK, DETECTR, and STOPCovid. CRISPR-Cas biosensing technology has been widely applied to point-of-care testing (POCT) by targeting recognition of both DNA molecules and RNA Molecules.

## 1 Introduction

The CRISPR (Clustered Regularly Spaced Short Palindromic Repeats) system is a revolutionary technology that has transformed the field of genetic engineering (Wang and Doudna, 2023). Originally discovered in prokaryotes as an immune mechanism to clear foreign nucleic acids, CRISPR-Cas has been rapidly and extensively used in basic and applied research due to its strong ability for gene editing, regulation, and detection in eukaryotes (Liu et al., 2022; van Beljouw et al., 2023). The CRISPR-Cas system consists of two main components: the Cas protein, which acts as a molecular scissors to cut DNA at specific locations, and the guide RNA (gRNA), which directs the Cas protein to the target site (Liu et al., 2022; van Beljouw et al., 2023). By modifying the gRNA sequence, researchers can precisely target any gene of interest and introduce specific mutations or insertions. Essentially, CRISPR-Cas systems can be programmed to recognize specific nucleic acid sequences, such as those found in viruses or cancer cells. When the system encounters the target sequence, it triggers a response that can be detected and used for diagnosis (Weng et al., 2023). In recent years, the potential applications of CRISPR-Cas technology have expanded to include the diagnosis and treatment of various diseases (Dhainaut et al., 2022; Katti et al., 2022; Chavez et al., 2023; Musunuru, 2023; Wang and Doudna, 2023), including severe acute respiratory syndrome coronavirus 2 (SARS-CoV-2). SARS-CoV-2 is a highly infectious virus that causes COVID-19, a respiratory illness that has rapidly spread across the globe since its emergence in late 2019 (Carabelli et al., 2023). The development of effective diagnostic tools and treatments for SARS-CoV-2 is critical for controlling its spread and reducing its impact on public health.

In this review paper, we will provide an overview of the biology and mechanisms of CRISPR-Cas technology and its relevance to SARS-CoV-2 diagnosis and treatment. We will discuss how researchers have adapted CRISPR-Cas technology for use in detecting SARS-CoV-2 RNA from patient samples with high sensitivity and specificity. We will also explore how CRISPR-Cas technology can be used to develop new therapies for SARS-CoV-2 by targeting viral proteins or host factors essential for viral replication. Additionally, we will address some potential ethical concerns surrounding the use of CRISPR-Cas technology in combating diseases like SARS-CoV-2. Overall, this review paper aims to provide readers with a comprehensive understanding of how CRISPR-Cas technology can be leveraged to combat SARS-CoV-2 through both diagnostic testing and therapeutic interventions.

## 2 CRISPR-cas nucleic acid detection tool

### 2.1 Overview of CRISPR-Cas9 technology

**FIGURE 1:**
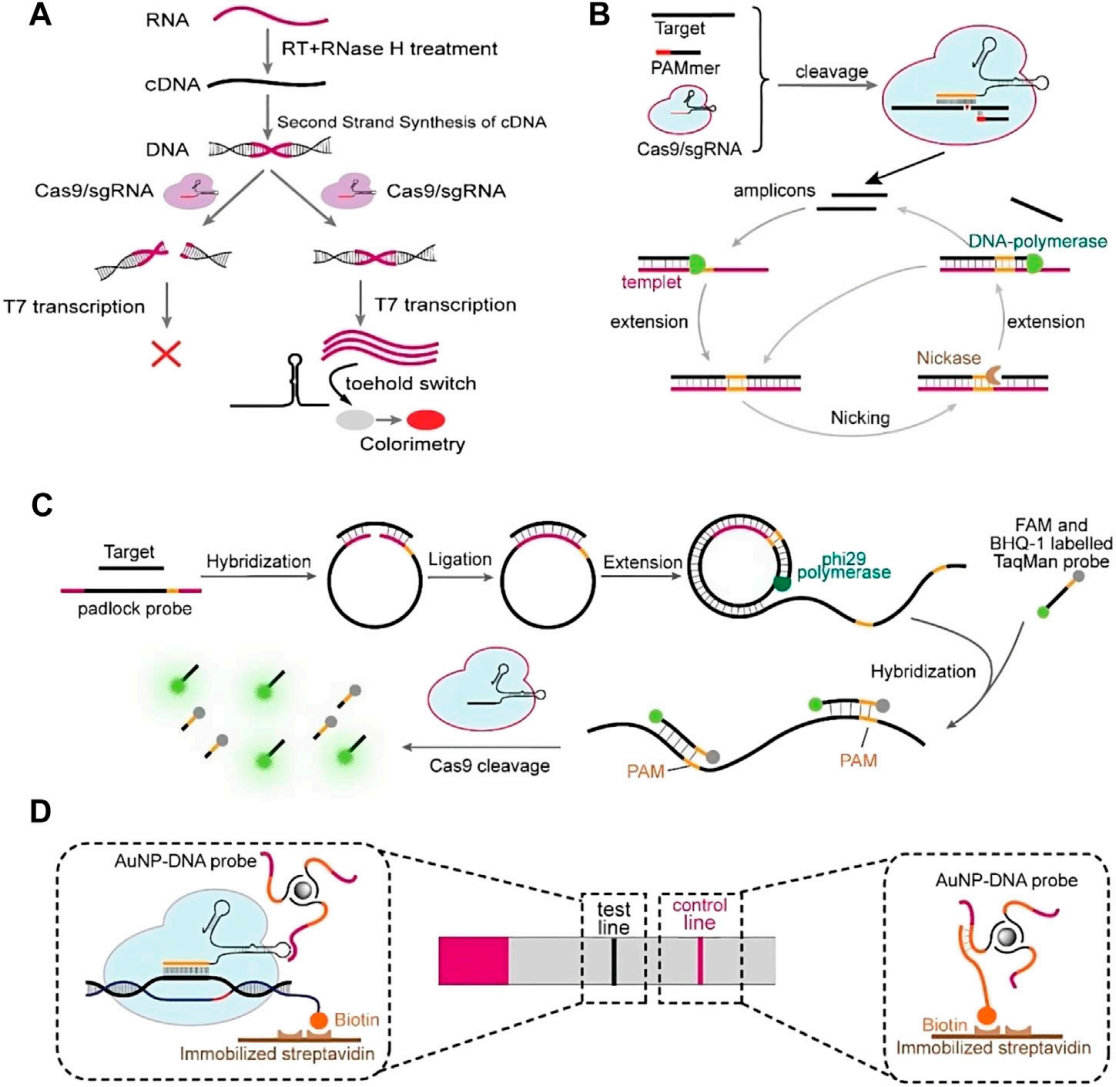
CRISPR-Cas9-mediated biosensing assays. **(A)** Cas9-based NASBACC assay that can distinguish single-base mismatches located in the PAM region. **(B)** CAS-EXPAR for ultrasensitive detection of single-stranded targets. **(C)** RACE for miRNA detection. **(D)** CASLFA is a Cas9-based lateral flow assay that enables fast and field-deployed detection. Reprinted with permission from Wang et al. (2020a). Copyright ^©^ 2020 Elsevier B.V. All rights reserved.

The CRISPR-Cas9 system is a type II CRISPR system containing HNH and RuvC domains. It uses Cas9 protein, trans-activating CRISPR RNA (tracrRNA), and CRISPR RNA (crRNA) to edit the target gene. Cas9 recognizes the protospacer adjacent motif (PAM) (Figure 1) and cleaves the target DNA. The CRISPR-Cas9 system can be combined with optical DNA mapping and DNA fluorescence *in situ* hybridization (FISH) to detect antibiotic resistance genes and methicillin-resistant *staphylococcus aureus* (MRSA), respectively (Guk et al., 2017; Yamano et al., 2017). Moreover, the dead Cas9 system can be utilized to regulate gene transcription and bind DNA without cleaving it. *In vitro* DNA detection systems for dead Cas9 have been developed, detecting *mycobacterium tuberculosis* DNA with high specificity and sensitivity. However, inaccurate target sequence recognition during the cutting process may lead to mutation, and the crRNA-tracrRNA complex required for target nucleic acid recognition is more complicated than other recognition methods (Muller et al., 2016).

### 2.2 Overview of CRISPR-Cas12 technology

The Cas12a protein, a type V CRISPR-related protein, guided by RNA, has an incidental cleavage activity and contains the RuvC domain. Under the guidance of crRNA, the target DNA binds to double-stranded DNA (dsDNA) in a targeted way, forming a ternary complex and activating the trans-cleavage activity of the non-specific Single-stranded DNA (ssDNA) (Chen et al., 2018; Liu et al., 2021a). Combining Cas12a with isothermal amplification, the DETECTR system performs isothermal amplification on target DNA and uses Cas12a to amplify the target DNA under the guidance of crRNA (Li et al., 2018a; Petri and Pattanayak, 2018). The quenched ssDNA reporter gene is cleaved to generate a fluorescent signal. The system has been applied to transgenic detection, achieving rapid and sensitive detection of transgenic products and their products. The HOLMES system, using fluorescence reporting and CAS12A for detecting target DNA and RNA, can detect single nucleotide polymorphisms and DNA and RNA viruses (Li et al., 2018a). For more convenient detection, the OCTOPUS platform based on the CAS12A cutting mechanism was developed, providing a guarantee for food safety (Wang et al., 2020b). The CRISPR-Cas12a biosensor can be used to detect Urasi-DNA glycoenzyme (UDG) and T4 polynucleotidase kinase (T4 PNK) and can determine real samples, including single-cell oxygenation and human plasma (Du et al., 2021). Cas12b, another commonly used nucleic acid protein, can be used for effective gene editing in mammals and humans. It also shows the same trans-cleavage activity on DNA. Heuristic Learning of Meanings and Explanations System version 2 (HOLMESv2) system, using Cas12b, can specifically distinguish single nucleotide polymorphisms, providing the potential for ultra-sensitive and specific DNA detection (Li et al., 2019a). A highly sensitive specific lateral flow biosensor was developed by integrating Cas12a and Cas12b effectors with Loop-mediated Isothermal Amplification (LAMP) for detecting *Pseudomonas aeruginosa* (Mukama et al., 2020).

### 2.3 Overview of CRISPR-Cas13 technology

Cas13, a type VI CRISPR system-related protein, falls into the RNA-guided and RNA-targeted RNase protein family (Ali et al., 2018). It contains two divergent higher eukaryotes and prokaryotes nucleotide-binding (HEPN) domains that generate multiple cleavage sites in single-stranded regions of target RNA with protospacer flanking sites (Abudayyeh et al., 2016). Cas13 boasts the ability to implement RNA editing and extensive applications in nucleic acid detection and disease diagnosis. The SHERLOCK platform, combined with Cas13a, can detect RNA molecules, achieving single-molecular sensitivity and single-base specificity for viral nucleic acid (Gootenberg et al., 2017; Myhrvold et al., 2018; Kellner et al., 2019). A high-sensitivity electrochemical biological sensitization platform for miRNA was established by combining CRISPR-Cas13a with catalytic hairpin assemblies (Cui et al., 2021). The SHERLOCKv2 platform, increasing signal sensitivity, was developed by combining Cas13a with the auxiliary CRISPR-III-related nuclease Csm6 (Niewoehner and Jinek, 2016). The CRISPR-Cas13 system has full molecular sensitivity and single-base detection specificity, providing technical support for epidemiological monitoring and pathogen detection in areas with underdeveloped infrastructure. The DETECTR system is more convenient for DNA detection due to the elimination of the *in vitro* transcription process (Li et al., 2018a; Petri and Pattanayak, 2018).

### 2.4 Overview of CRISPR-Cas14 technology

Cas14, which is the smallest type II CRISPR effector that targets ssDNA and can be activated without PAM, shows different fidelity mechanisms from Cas12a after recognition (Savage, 2019). It has non-specific DNase activity, and DETECTR-Cas14, an ssDNA detection platform, was developed based on this property (Aquino-Jarquin, 2019). Compared to DETECTR, which is related to Cas12a, DETECTR-Cas14 shows higher fidelity in distinguishing ssDNA substrates and can be used for DNA single nucleotide polymorphism genotyping and detection and diagnosis of ssDNA pathogens (Li et al., 2018a). The CRISPR-Cas14 system can be used in combination with the heating clinical samples to remove nuclease (HUDSON) method for rapid virus detection and has been successfully applied for parvovirus detection of human bocavirus (HBoV1) (Gootenberg et al., 2017; Aquino-Jarquin, 2019). Additionally, the accuracy of this new diagnostic technique has been demonstrated in detecting the human E3 ubiquitin protein ligase (HERC2) gene (Li et al., 2018a). However, the application of Cas14 in diagnostics is more complex as additional steps are required to generate ssDNAaCas14 from the dsDNA target prior to detection, and its advantages have not been clearly demonstrated to date (Savage, 2019). Comparison of various CRISPR detection systems is shown in Table 1.

**TABLE 1:** Comparison of common CRISPR systems.

Effector	Domain	Target type	Signal RNA	Detection platform	Ampli-fication	Quanti-tative	Time	Sensitivity	Portable
Cas9	HNH, RuvC	DNA	tracrRNA, crRNA	-	CAS-EXPAR	No	<1 h	aM	Yes
Cas12a	RuvC	DNA	crRNA	HOLMES, DETECTR	HOLMES: PCR; DETECTR: RT-PCR, RPA	No	1 h	aM	HOLMES: yes; DETECTR: no
Cas12b	RuvC	DNA	crRNA	HOLMESv2	LAMP, RT-LAMP	Yes	1 h	aM	Yes
Cas13a	HEPN	RNA	crRNA	SHERLOCK	RPA	No	2 h	aM	Yes
Cas14	RuvC	ssDNA	tracrRNA, crRNA	DETECTR	RPA	No	1 h	aM	Yes

RPA, recombinase polymerase amplification.

### 2.5 CRISPR nucleic acid amplification detection technology

CRISPR detection methods require nucleic acid amplification, and while standard PCR amplification is not portable and takes a long time, other isothermal amplification methods such as recombinase polymerase amplification (RPA) and LAMP have been commonly used. These have been combined with CRISPR to develop detection platforms like DETECTR and SHERLOCK (Niewoehner and Jinek, 2016; Li et al., 2018a). Other amplification methods include DNA-amplified multimer reporter (DAMR), HOLMESv2, rolling circle amplification (RCA), and NASBA (Pardee et al., 2016; Li et al., 2019a; Wang et al., 2020c). HOLMESv2 system is combined with LAMP amplification, and under constant temperature conditions, Cas12b detection can be combined with LAMP amplification and undergo bisulfite treatment to accurately quantify the degree of target DNA methylation (Li et al., 2019a). RCA is a highly specific isothermal gene amplification method that can be performed at room temperature, and can assist CRISPR-Cas9 in detecting multiple extracellular vesicle small RNAs (Wang et al., 2020c). NASBA is capable of accurately distinguishing closely related Zika virus strains when combined with CRISPR-Cas9 (Pardee et al., 2016).

### 2.6 CRISPR colorimetric readout detection system

Chromaticity sensors are preferred in CRISPR detection because they allow direct visualization of results. Lateral-flow analysis (LFA) and CRISPR-Cas9-mediated lateral flow nucleic acid assay (CASLFA) are two methods that have been used to identify various targets (Wang et al., 2020d). A colorimetric assay based on colloidal Au nanoparticles (AuNP) and platinum nanoparticles (PTNPs) has also been adopted to detect multiple cancer gene mutations in serum using their volumetric bar chips (Yuan et al., 2020).

### 2.7 CRISPR and signal output system: Rapid DNA detection without amplification

This system combines CRISPR cutting with an electronic signal output to accurately observe changes in results at low concentrations without target amplification. By using graphene-based field-effect transistor (gFET) and nanopore sensors, CRISPR-dCas9 affects the output of electronic signals by identifying target sequences, and detects report DNA fragments by trans-cutting of Cas12a, which effectively solves the detection time problem of low concentration target DNA (Nouri et al., 2020). In addition, electrochemical sensing system can obtain high transduction signal using the trans-cleave activity of Cas12a to indicate the presence of target DNA *via* current difference (Dai et al., 2019).

## 3 CRISPR-cas technology used in nucleic acid detection

### 3.1 Principle of nucleic acid detection technology based on CRISPR-cas system

CRISPR-Cas system has three stages: adaptation, expression, and interference. In the detection technology based on CRISPR-Cas system, these stages are used for targeted modification to achieve the detection of specific targets. Single-guide RNA (SGRNA) is designed to recognize the target sequence, and then CAS protein cleaves the target sequence to form a specific double-strand break (DSB) (Aman et al., 2020). Different CAS proteins have different mechanisms in nucleic acid detection (Aman et al., 2020), with Cas9 relying on the ability to specifically recognize target sequences and specific sgRNA designed for target detection (Li et al., 2019b; Aman et al., 2020). Nucleic acid detection methods based on Cas12, Cas13, and Cas14 rely on the accessory cleavage activity of Cas protein (Zetsche et al., 2015; Harrington et al., 2018; Aman et al., 2020). When Cas protein forms an effect complex with sgRNA and target sequence, its accessory cleavage activity is activated, and the labeled ssDNA or ssRNA reporter gene is cleaved, thus releasing a signal to achieve the detection effect (Li et al., 2019b).

### 3.2 Application strategies of CRISPR-cas technology in nucleic acid detection

CRISPR-Cas system has been applied in genome editing and extensively studied as a new biotechnology (Cong et al., 2013). Different CAS proteins are selected for different pathogenic microorganisms to be detected (Harrington et al., 2018). The nucleic acid detection based on Cas9 mainly relies on the ability to specifically recognize target sequences (Cong et al., 2013). Nucleic acid detection methods based on Cas12, Cas13, and Cas14 rely on the accessory cleavage activity of Cas protein (Shmakov et al., 2015; Zetsche et al., 2015). Among known Cas proteins, only Cas13a can directly target RNA viruses. If other types of Cas protein are selected in practice, the RNA molecule to be detected needs to be reverse transcribed into DNA before the corresponding detection method can be established (Shmakov et al., 2015; Zetsche et al., 2015).

#### 3.2.1 Application of Cas9 in nucleic acid detection

A paper-based sensor (NASBA/CRISPR Cleavage, NASBACC) was developed for the detection of Zika virus (Pardee et al., 2016). Two nuclease domains of Cas9 were mutated to obtain dCas9 (nuclear-deactivated Cas9) with no shearing ability, and a nucleic acid detection method based on luciferase modified dCas9 protein was developed for the detection of *Mycobacterium tuberculosis* (Gilbert et al., 2013; Qi et al., 2013; Zhang et al., 2017). Additionally, a RCA-CRISPR-HRP (RCH) detection method based on CRISPR-Cas9 was established to achieve low-cost and efficient detection of fM-level and single-base differential miRNA (Qiu et al., 2018).

#### 3.2.2 Application of Cas12 in nucleic acid detection

An accurate and rapid detection method was developed based on the accessory cleavage activity of Cas12a (Chen et al., 2018). The dsDNA of HPV was extracted from anal swabs and amplified by recombinase polymerase amplification (RPA). Then Cas12a-crRNA complex and ssDNA probe capable of fluorescence quenching were added into the reaction system (Chen et al., 2018). This detection method, called DETECTR, is a highly specific detection method that can accurately detect HPV16 and HPV18 from a variety of different human papillomavirus (HPV) subtypes (Chen et al., 2018; Wang et al., 2019). In order to improve the detection sensitivity and specificity, and reduce the detection time and contamination in the reaction process, a detection method called Cas12aVDet was proposed based on DETECTR in subsequent studies (Wang et al., 2019). Chen et al. established a portable CRISPR–Cas12-based field-deployable system for rapid detection of synthetic DNA sequence of the monkeypox virus (MPXV) genome (Chen et al., 2023a). This system takes advantage of CRISPR-Cas12’s high selectivity and the isothermal DNA amplification capabilities of recombinase polymerase amplification (Chen et al., 2023a). It is capable of identifying both the presently circulating MPXV clade and the original clades. Using a microtiter plate reader, they achieved a limit of detection (LoD) of 22.4 a.m. (13.5 copies/µl), while the visual LoD for the system is 75 a.m. (45 copies/µl) in a two-step assay, further decreasing to 25 a.m. (15 copies/µl) in a one-pot system (Chen et al., 2023a). Gul et al. reviewed the traditional and emerging nucleic acid detection approaches, immunodiagnostics, whole-particle detection, and imaging-based MPXV detection techniques, helping researchers to develop novel techniques for the diagnosis of MPXV (Gul et al., 2022a).

#### 3.2.3 Application of Cas13 in nucleic acid detection

A detection platform based on CRISPR-Cas13a system was designed by researchers according to the properties of Cas13a, which was named SHERLOCK (specific high sensitivity enzymatic reporter unlocking) (Gootenberg et al., 2017). RNA coupled with the fluorescent reporter molecule is cleaved, and the generated fluorescent signal is amplified by enzymatic activity, thus enhancing the detection sensitivity. HUDSON was introduced by Myhrvold et al., in 2018 to release and protect viral nucleic acids from clinical samples, avoiding the need for nucleic acid extraction in molecular diagnosis (Myhrvold et al., 2018). A sensitive, specific test platform was developed by combining HUDSON with SHERLOCK, with which Zika virus (ZIKV) and Dengue virus (DENV) can be detected directly from body fluids (urine, saliva, serum, plasma and whole blood) with limited samples and equipment requirements, and the results can be obtained within 1–2 h. The nucleic acid detection technology based on CRISPR-Cas13 boasts higher sensitivity, better specificity, convenience, and low cost without expensive equipment and professional operators compared with traditional PCR technology (Hadidi, 2019; Aman et al., 2020; Wang et al., 2020e).

#### 3.2.4 Application of Cas12/Cas13 in nucleic acid detection

Different CAS proteins can be used in combination based on the difference in their target substrates. An upgraded version of SHERLOCK (SHERLOCKv2) was developed to detect three ssDNA targets and one dsDNA target in a single reaction. Seven CRISPR-Cas13a and/Cas13b enzymes were biochemically identified, and three Cas13 proteins with different cleavage preferences were selected to be used in combination with Cas12a and RPA (Gootenberg et al., 2018).

#### 3.2.5 Application of Cas14 in nucleic acid detection

Recently, the CRISPR-Cas14 system has been discovered by researchers, which contains Cas14a that can target ssDNA sequences and has accessory activity mediated by target activation (Harrington et al., 2018). With this system, different ssDNA can be targeted in multiple ways, so nucleic acid detection technology and platform can be developed for viruses with ssDNA as genetic material (Aquino-Jarquin, 2019). This system has the potential to be effectively applied in the field of molecular diagnosis (Harrington et al., 2018; Aquino-Jarquin, 2019).

### 3.3 Advantages and limitations of nucleic acid detection based on CRISPR-cas technology

Nucleic acid detection based on CRISPR-Cas technology has the potential to revolutionize global public health by enabling rapid, sensitive, stable, and accurate real-time field detection (Sashital, 2018). The detection systems established are also different (Table 2). It offers many advantages over traditional detection methods, such as simple operation, easy development, high resolution for single base mutation, and the ability to detect down to the level of single molecule detection. Additionally, it does not require expensive instruments, can be read *via* visualization, and can achieve quantitative detection and multiple detection. However, there are limitations to this technology (Kieper et al., 2018). One such limitation is that there are specific requirements for crRNA to guide Cas protein to recognize and cleave targets. Specifically, base pairing between crRNA and target sequences, as well as a suitable PAM sequence at the 3’ end of the target sequence, are needed. Further research is needed to develop appropriate detection strategies for quantitative detection and simultaneous detection of multiple targets (Pardee et al., 2016). Therefore, it is important to consider and evaluate the possible advantages and disadvantages of these technologies, such as whether different DNA targets or RNA targets need to be detected simultaneously for the nucleic acid types of the samples to be tested, whether PAM sequences exist in the sequences, and whether there are different requirements for the output mode and sensitivity, so as to select the optimal detection strategy. In conclusion, nucleic acid detection based on CRISPR-Cas technology has great potential to bring more convenient, efficient, low-cost, and practical diagnostic tools for diseases caused by pathogenic microorganism infection around the world (Sashital, 2018). Despite the limitations of this technology, further research and development can lead to more innovations and possibilities for nucleic acid detection technology.

**TABLE 2:** Applications of nucleic acid detection based on CRISPR-Cas systems.

Detection system	Signal output	Target	Sensitivity	Advantage	Combined technology
dCas9	Fluorescence	Methicillin-Resistant *Staphylococcus Aureus*	10 cfu/mL	Without PCR amplification or gene purification steps	FISH
Cas9	Fluorescence	*Listeria* Monocytogenes	0.82 a.m.	Fast	EXPAR
Cas13a	Fluorescence	*Vibrio* Parahemolyticus	Molecule/Reaction	Lower cost of enzyme	RAA
DETECTR	Fluorescence	HPV16/18	aM	Single-base mismatch specificity, without transcription	RPA
SHERLOCK	Fluorescence	Virus, bacteria, genotype human DNA	zM	High sensitivity and single-base mismatch specificity	T7 transcription and RPA
SHERLOCKv2	Fluorescence	Virus, bacteria, genotype human DNA	10 copies of DNA	Multiplexing, visual readout	RPA
Cdetection	Fluorescence	HPV16/18	aM	Single-base mismatch specificity	RPA
HOLMES	Fluorescence	JEV, RPV	aM	Single-base mismatch specificity, without transcription	PCR

## 4 Nucleic acid detection by CRISPR derivation technology

The CRISPR system was originally discovered in prokaryotes as a unique immune mechanism to clear foreign nucleic acids. Its strength in gene editing, regulation, and detection in eukaryotes has made it a popular tool in basic and applied research (Wright et al., 2016).

### 4.1 SHERLOCK

Cas13 belongs to a type VI CRISPR system, which is distinct from Cas9 in that it is an RNA cleaving nuclease. CRISPR-Cas13 has been used for the detection of nucleic acids of the Ebola and Zika viruses, as well as N1 methyladenosine (M1A) in RNA (Qin et al., 2019a; Chen et al., 2019; Freije et al., 2019). SHERLOCK (Specific High Sensitivity Enzymatic Reporter Unlocking) is a novel nucleic acid detection method developed by Zhang et al. based on the use of Cas13a to cleave the target sequence and surrounding RNA, followed by the introduction of single-stranded RNA with a fluorescent reporter group (Myhrvold et al., 2018). After Cas13a cleaves the target sequence, the single-stranded RNA with the fluorescent reporter group is cleaved to separate the fluorescent group from the quenched group, and the detection is completed by observing the fluorescence signal. This method consists of two steps: RPA and T7 transcription *in vitro*, which allows for the amplification and extraction of virus nucleic acid in the samples to be detected. However, this technology is currently only suitable for qualitative detection due to its low accuracy.

**FIGURE 2:**
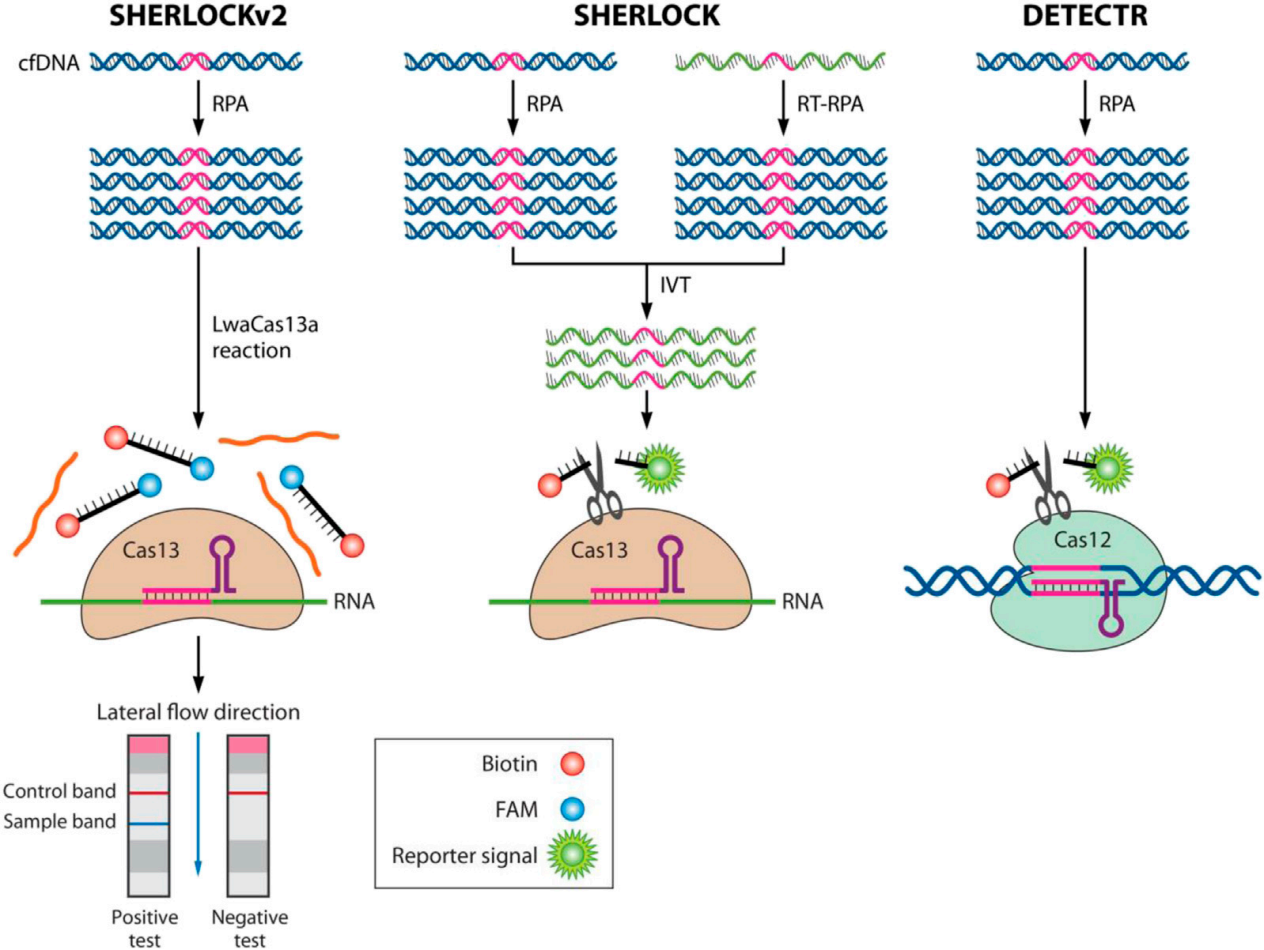
CRISPR-Cas technologies for nucleic acid detection in SHERLOCKv2, SHERLOCK, and DETECTR assays. In the absence of its nucleic acid target, the Cas nuclease is inactive. When binding to its guide crRNA to a related target (RNA for Cas13a, ssDNA or dsDNA for Cas12a), the nuclease is activated, leading to catalytic cleavage of off-target nucleic acids (RNA for Cas13a, ssDNA for Cas12a). This collateral nuclease activity is turned into an amplified signal by providing reporter probes with a fluorophore (green) linked to a quencher (white) by a short oligonucleotide (black). (Left) Schematic of SHERLOCKv2, with direct detection of viral infection (for example) in bodily fluids. (Middle) Schematic of the SHERLOCK system. Nucleic acid is extracted from clinical samples (for example), and the target is amplified by recombinase polymerase amplification (RPA) with either RNA or DNA as the input (reverse transcriptase recombinase polymerase amplification [RT-RPA] or RPA, respectively). RPA products are detected in a reaction mixture containing T7 RNA polymerase, Cas13, a target-specific crRNA, and an RNA reporter that fluoresces when cleaved. (Right) Schematic of the DETECTR system. Reprinted with permission from Mustafa and Makhawi (2021). Copyright ^©^ 2021 Mustafa and Makhawi.

Zhang et al. made improvements to SHERLOCK in SHERLOCK V2 on the basis of the original method. The updated method includes four improvements (Gootenberg et al., 2018; Myhrvold et al., 2018): (1) Four-channel, single-reaction, and multi-channel detection with four orthogonal CRISPR enzymes (LwaCas13a, PsmCas13b, CcaCas13b, ASCAS 12a); (2) Quantitative detection of viral nucleic acid load as low as 2 a.m.; (3) Cas13 was combined with Csm6 to increase the sensitivity of the detection signal by 3.5 times (Garcia-Doval et al., 2020); (4) Visual reading with lateral flow test strips. The upgraded SHERLOCK V2 overcomes the dependence on fluorescent detection equipment and is capable of performing quantitative and large-scale detection for a variety of diseases (Figure 2).

The detailed operating procedure of applying SHERLOCK to detect SARS-CoV-2 was released, and on 7 May 2020, the first SARS-CoV-2 nucleic acid detection product based on SHERLOCK V2 technology, Sherlock CRISPR SARS-CoV-2 kit, was authorized by the FDA of the United States for emergency use. This is the first time that the FDA has approved a SARS-CoV-2 detection product based on CRISPR gene editing technology, and also the first time that the technology has been authorized for use in the detection of infectious diseases (Shihong Gao et al., 2021).

### 4.2 DETECTR

Cas12a (also called Cpf1) is an RNA-guided nuclease that cuts DNA at specific sites. It is used for gene editing, similar to CRISPR-Cas9 (Swarts, 2019). Doudna et al. developed a nucleic acid detection method called DETECTR (DNA Endonuclear Targeted Crispr Trans Reporter) that uses Cas12a′s characteristics (Chen et al., 2018). They introduced a single-stranded DNA molecule with a fluorescent reporter into the reaction system (Chen et al., 2018). When Cas12a cuts the target sequence, the single-stranded DNA connected to the fluorescent reporter will also be cut, causing the reporter to emit fluorescence and detect the target sequence.

Broughton et al. developed a DETECTR-based detection kit for SARS-CoV-2 (Broughton et al., 2020). The kit only took 45 min to complete and had a positive predictive agreement of up to 95%. Doudna et al. are working with GlaxoSmithKline to develop a disposable household SARS-CoV-2 test kit using this technology.

Wang et al. developed a SARS-CoV-2 nucleic acid detection method called CRISPR-Cas12a-NER based on CRISPR-Cas12a (Wang et al., 2020f). It can detect samples with medium to low 10 virus copies per microliter. Quenching green fluorescence is marked on the ssDNA reporter gene, which can be observed with the naked eye under blue light of 485 nm. The whole detection process takes 45 min. However, this method is still in clinical trials and has not yet gained market access.

### 4.3 STOPCovid

Cas12b (also known as C2C1) is a small nuclease that is stable, long-lasting, and resistant to high temperatures (Teng et al., 2018). SHERLOCK is a SARS-CoV-2 nucleic acid detection technology that involves multiple steps and increases the risk of environmental contamination. To address this issue, Zhang et al. developed a simpler method called STOPCovid (SHERLOCK Testing in One Pot, STOPCovid) (Joung et al., 2020a). This method detects the virus by amplifying viral RNA using LAMP technology and then cleaving the RNA with Alicyclobacillus acidiphilus Cas12b (AapCas12b). An upgrade to STOPCovid by Zhang et al. introduced magnetic beads to simplify the extraction process of viral RNA and increase the sensitivity of the reaction. This updated method, STOPCovid.V2, has a sensitivity and specificity of 93.1% and 98.5%, respectively (Joung et al., 2020b). However, it is still in the pre-clinical testing stage. CRISPR technology has a high detection sensitivity and strong specificity, making it a promising nucleic acid technology for detecting SARS-CoV-2 infection. However, throat swab sampling is still required, and the quality of the detection kit may affect its accuracy.

## 5 Applications of CRISPR-cas biosensor technology to point-of-care testing (POCT)

POCT is a medical testing approach that allows for rapid and convenient diagnostic testing at or near the point of patient care. This type of testing can be performed by non-laboratory trained personnel and can provide real-time results, allowing for faster diagnosis and treatment. The integration of POCT with CRISPR technology holds the promise of significantly transforming the diagnostic testing landscape. By leveraging the innovative capabilities of CRISPR-based POCTs, the precision and expediency of diagnosing a diverse array of diseases and conditions could be substantially enhanced. Furthermore, the implementation of such tests could be expanded to encompass a broader range of environments, encompassing rural or isolated regions where conventional laboratory-based diagnostics may be inaccessible (Chen et al., 2023b).

### 5.1 Work principle of CRISPR-Cas biosensing technology

The CRISPR-Cas system is divided into two categories: Class I and Class II. Class I uses a complex of multiple proteins and guide RNA to edit genes, while Class II only uses a single protein effector and guide RNA to edit genes (Wright et al., 2016). Cas12a, Cas3a, and Cas14 are examples of Class II and are useful in nucleic acid detection due to their non-specific cutting activity after binding to the target gene (Chen et al., 2018; Harrington et al., 2018). This technology has higher detection sensitivity and specificity compared to traditional nucleic acid detection methods.

#### 5.1.1 CRISPR-cas biosensor system for targeted recognition of DNA molecules

Type V Cas12a of CRISPR-Cas system type II is an RNA guided endonuclease that cleaves specific DNA. Studies show that after the formation of ternary complex of Cas12a, crRNA and target DNA, it produces non-specific cutting fluorescence quenching of single stranded DNA reporting group and emit fluorescence signal (Li et al., 2018b). Li et al. developed the high-fidelity, optimized, ligand-activated, dna cleavage enzyme (HOLMES) technology for rapid detection of target DNA and RNA. Their detection sensitivity can reach the level of single molecule (Li et al., 2018a). The DETECTR nucleic acid detection technology based on Cas12a combines RPA technology to amplify the target gene, which can detect HPV and distinguish HPV16 and HPV18 strains (Chen et al., 2018). To directly detect RNA, Li et al. established the HOLMESv2 nucleic acid detection technology by combining the LAMP technology with Cas12b digestion of the target gene (Li et al., 2019a). Similarly, Cas12b protein was used to realize high sensitivity and single base specific detection at single molecular level (Teng et al., 2019).

Type V Cas14a, belonging to the CRISPR-Cas system of type II, has been found to have non-specific nucleic acid molecular cutting activity after activation (Harrington et al., 2018). Unlike Cas12a, it does not require the PAM to recognize target genes. DETECTR-Cas14a technology based on the Cas14a system is more specific in detecting monobasic groups, but it can only recognize single-stranded DNA molecules. Therefore, after target gene amplification is complete, DNA double strands must be unlocked to achieve nucleic acid detection.

#### 5.1.2 CRISPR-cas biosensor system for targeted recognition of RNA molecules

Cas13a is a RNA-guided endonuclease that targets RNA, and can cleave single-stranded RNA under the guidance of crRNA. It has non-specific RNA cleaving activity. Using fluorescence quenching, nucleic acid detection can be achieved. SHERLOCK technology, based on Cas13a, detects target DNA and RNA by first amplifying the target gene and then transcribing the amplified product *in vitro* to serve as the target RNA molecule (Gootenberg et al., 2017). This method can detect single strains of Zika virus (ZIKV) and dengue virus (DENV), perform SNP typing of human genes, and detect tumor DNA mutations. SHERLOCKv2 detection technology, which combines Cas12a, Cas13 and type III CRISPR effect nuclease Csm6, enables highly sensitive and multi-channel multiple nucleic acid detection. In combination with HUDSON and lateral flow test strip detection technology, SHERLOCKV2 provides highly sensitive and independent nucleic acid detection of ZIKV and DENV (Gootenberg et al., 2017; Myhrvold et al., 2018).

### 5.2 Steps of POCT using CRISPR-cas biosensor technology

POCT technology allows for fast test results without the need for complex experimental instruments or special laboratory sites (Wiencek and Nichols, 2016). With the increasing demand for on-site diagnosis and the popularity of nucleic acid detection, POCT has rapidly developed. It is important in the field of public health and has expanded to government import and export trade protection. The current on-site nucleic acid detection method involves the use of the CRISPR-Cas biosensor system, which includes rapid nucleic acid extraction, target gene amplification, Cas digestion, and crRNA preservation.

#### 5.2.1 Rapid extraction of nucleic acid

Pathogen detection involves sample collection and nucleic acid preparation, typically by mechanical, enzymatic, or chemical methods. However, these methods require equipment that may not be available for on-site detection. New methods have been developed to simplify nucleic acid extraction, such as HUDSON (Myhrvold et al., 2018) or extracting RNA within 5 min (Joung et al., 2020b). These methods have been successful in detecting viruses like ZIKV, DENV, and SARS-CoV-2 in urine or saliva. For African Swine Fever Virus (ASFV) detection, Wang et al. incubated serum samples at room temperature for 3 min to release the virus DNA (Wang et al., 2020g). These simpler extraction methods are useful for rapid on-site nucleic acid detection with CRISPR-Cas biosensor systems.

#### 5.2.2 Isothermal nucleic acid amplification

Sensitive nucleic acid detection relies on Cas proteins and nucleic acid amplification methods, such as PCR (Zhu et al., 2020). However, PCR requires a large instrument and is not suitable for on-site detection. Isothermal amplification techniques like RPA or LAMP are more suitable for on-site detection (Chen et al., 2018). LAMP uses 4 or 6 specific primers for target gene regions to achieve nucleic acid amplification (Notomi et al., 2000), Compared to RPA, LAMP has more advantages, including direct amplification using DNA or RNA as templates, which makes it easier to detect RNA polymerase (RNAP). Using DETECTR technology to detect SARS-CoV-2 only requires Reverse-transcription LAMP (RT-LAMP) one-step nucleic acid amplification and Cas digestion to detect fluorescence signal (Broughton et al., 2020).

#### 5.2.3 Freeze dry storage of cas protein and crRNA

Proteins and RNA can be easily degraded, which makes it necessary to store them at −20°C to prevent inactivation. However, this can be a problem for on-site nucleic acid detection. Researchers have explored using a vacuum freeze dryer to create a freeze-dried powder of reaction reagents like Cas13a protein and crRNA for long-term preservation (Kellner et al., 2019).

#### 5.2.4 Interpretation method of test results

The CRISPR-Cas biosensor system identifies target genes and then cuts the reporter gene non-specifically. Lateral flow test strips and fluorescence visual detection technology are currently combined to detect nucleic acid in the field. The detection principle of lateral flow strips is to use DNA or RNA reporter genes with fluorescein and biotin tags at the ends (Qin et al., 2019b). When Cas protein and crRNA recognize the target gene, they cut the reporter gene to release fluorescein. Fluorescein then moves along the test strip and sample lines appear to indicate the detected target molecules. SHERLOCK technology has been used by Gootenberg et al. and Joung et al. to detect SARS-CoV-2, ZIKV, DENV and other viruses (Gootenberg et al., 2017; Joung et al., 2020b). Wang et al. achieved high sensitivity detection of ASFV in CRISPR-Cas12a LFD (Wang et al., 2020g), while Broughton et al. achieved 45 min detection of SARS-CoV-2 using DETECTR technology (Broughton et al., 2020).

Fluorescence signal can enable on-site nucleic acid detection. He et al. developed a portable fluorescence signal acquisition instrument and combined Cas12a technology to detect ASFV with high throughput (He et al., 2020). Wang et al. developed Cas12aVDe technology, which only requires blue light to detect light quenching report genes for screening and comparison (Wang et al., 2019). They established a new technology for enhanced fluorescence visual nucleic acid detection and successfully detected ASFV (Xie et al., 2022). Li et al. modified the reporting group using ionic gold nanoparticles (AuNPs), and established a nucleic acid detection technology that can detect grape vine erythema virus by directly observing the color change of the reaction solution with the naked eye. CRISPR-Cas biosensor system has been greatly promoted in on-site nucleic acid detection through optimization of the results interpretation method (Li et al., 2019c).

### 5.3 Strategies of appliying CRISPR-cas biosensor technology to POCT

#### 5.3.1 Detection strategy of target nucleic aid amplification before CRISPR-cas recognition

CRISPR/dCas9 is a recognition probe that separates nucleic acids from complex samples. Paired dCas9 (PC) technology uses two dCas9 proteins to detect adjacent sequences with luciferase domains, generating detectable fluorescence signals (Zhang et al., 2017). Uygun ZO et al. used CRISPR/dCas9 and electrochemical impedance spectroscopy (EIS) to detect circulating tumor DNA (ctDNA) and mutations in exon 9 of PIK3CA. The device has fast detection speed but a sensitivity of only 0.65 nmol/L (Uygun et al., 2020).

#### 5.3.2 Detection strategy of target nucleic acid amplification before CRISPR-cas cleavage

CRISPR-Cas recognition is used to detect specific sequences. In 2016, Pardee et al. developed NASBACC (for nucleic acid sequence-based amplification (NASBA)-CRISPR cleavage) technology, which combines NASBA technology, a toehold switch RNA sensor, and CRISPR-Cas9 system to detect Zika virus typing (Pardee et al., 2016). During the detection process, the RNA is introduced with a T7 transcriptional promoter and a sequence triggered by the toehold switch in primer design through NASBA technology. The amplification process cuts the dsDNA product into two segments by Cas9. Before sensor H is activated, the translation of the LacZ gene is blocked by isolating the ribosome binding site and the starting codon cis. When the toehold switch is combined with the complementary trigger RNA, RBS and the start codon are released, activating LacZ gene translation, and expression of β-Galactosidase. However, NASBACC’s reaction time is between 90–120 min, which requires denaturation steps and the efficiency of RNA amplification in the range of 120–250 bp is low (Zaghloul and El-Shahat, 2014). Therefore, it is less used in the existing POCT combined with CRISPR-Cas.

#### 5.3.3 Detection strategy of first using CRISPR-cas targeted cleavage and then nucleic acid

After CRISPR-Cas cleavage, nucleic acid amplification removes abundant interfering nucleic acids and targets rare and low abundance nucleic acid targets. Huang et al. developed a CRISPR-Cas9 triggered isothermal exponential amplification reaction (CAS-EXPAR) that detects DNA targets with atomole (aM) sensitivity and monobasic specificity in 1 h (Huang et al., 2018). The system detects DNA methylation and the hly mRNA of *Listeria* monocytogenes. The CasEXPAR technology first combines Cas9 with sgRNA to form a complex, identifies target sequences with customized PAM sequences, and cuts Cas9 at specific sites to generate target fragment X. Fragment X is used as a primer to combine with the amplification template, and under the action of DNA polymerase, complete double stranded DNA is synthesized. NEase introduces a cut on the double stranded DNA by recognizing the cleavage site. DNA polymerase extends again at the cut to form double stranded DNA and replaces the target fragment. The replaced target fragment can be used as a primer to start DNA synthesis, forming a circular amplification process that generates a large number of double stranded DNA products detectable with fluorescent dyes.

CRISDA technology uses CRISPR effectors and unique conformational rearrangements to identify target DNA, replacing the heating step in E-SDA and other technologies. A pair of programmed nCas9 proteins expand and cut the dsDNA region of interest (Zhou et al., 2018). With the help of SSB, polymerase extends the primer to stabilize the untwisted DNA. The newly formed chain is exponentially amplified and released after being cut by endonuclease. Combined with endpoint measurement mediated by peptide nucleic acid (PNA), this method detects various DNA targets in complex samples with aM sensitivity and single nucleotide specificity. The CRISDA reaction temperature is only 37°C, making it potential nucleic acid P0CT detection equipment. CRISPR-Cas9 targets and cleaves sequences of interest to produce shorter oligonucleotides for subsequent enrichment by amplification. This technique has been used to enrich target fragments for next-generation sequencing (Quan et al., 2019).

#### 5.3.4 Detection strategy of target nucleic acid amplification followed by CRISPR-cas trans cleavage

After nucleic acid amplification, the CRISPR-Cas system with trans cutting activity generates an amplified readout signal by recognizing specific nucleic acid sequences and randomly cutting nucleic acid like a particle shredder. This improves sensitivity and enables different report signals to expand the scope of application. The SHERLOCK detection platform uses Cas13, an RNA guided RNA endonuclease, to cleave specific ssRNA (Gootenberg et al., 2018). The system can detect up to 4 viruses at the same time and has a sensitivity 3.5 times higher than before (Myhrvold et al., 2018). The team also developed a 4-channel single reaction composite platform and combined all these developments with simple POCT, making it multi-channel, portable, fast, and quantitative as nucleic acid POCT. The system can directly detect Zika virus and dengue virus in urine and saliva samples of patients. In 2021, Liu et al. designed a tandem fast integrated nuclease detection platform FIND-IT (Liu et al., 2021b). This system uses Csm6 to amplify the effect of Cas13, and by adding a chemical modification activator that is stable and free from degradation, it improves the efficiency of Csm6 by 100 times compared with the original activator. The team integrated the optimized targeting RNA combination, so that when Cas13 is used in combination with Csm6 and its improved activator, as low as 31 cps/μ L SARS-CoV-2 RNA can be detected within 20 min.

Using crRNA, Cas12 can recognize and specifically cut dsDNA at the T-rich PAM site, while also cutting non-specifically at ssDNA. This method is combined with RPA and a detection platform called DETECTR to identify virus DNA without transcription. The temperature compatibility of RPA and CRISPR-Cas12 allows for POCT. HOLMES is a similar technology that uses Cas12a and asymmetric PCR to avoid the need for PAM. The amplification product of LAMP can be combined with CRISPR-Cas12b for isothermal amplification and signal generation in a program step.

Cas14 is a smaller RNA-guided nuclease that targets ssDNA. It requires complete complementarity of the sgRNA seed region to recognize the ssDNA substrate. Using Cas14a′s characteristics, Harrington et al. replaced Cas12a in DETECTR technology with Cas14a to create Cas14-DETECTR diagnostic tech (Chen et al., 2018; Harrington et al., 2018). The reaction principle involves modifying one primer used to amplify the target nucleic acid with thiophosphorylate (PT), and treating the dsDNA generated from amplification with T7 nuclease exonuclease to degrade one DNA strand not protected by PT, resulting in ssDNA. Cas14a endonuclease activity is stimulated, and the non-specific cleavage of the ssDNA fluorescence probe generates signals. This technique can distinguish single nucleotide polymorphism (SNP) in HERC2 gene related to eye color.

The SHERLOCK and DETECTR technologies require only portable equipment such as heating blocks or water baths, lateral flow devices, micro centrifuges, and pipettes. The improved “one pot” test of the SHERLOCK screening method for SARS-CoV-2, called STOPCovid, integrates sample processing, nucleic acid amplification, and detection using a new Cas orthologue, AapCas12b (Joung et al., 2020b). This Cas enzyme can maintain activity at the reaction temperature of the RT-LAMP process, making it well-suited for the temperature conditions of RT-LAMP. Pang et al. overcame the incompatibility between RT-LAMP and temperature conditions by ingeniously placing RT-LAMP and CRISPR-Cas12a reagents in the same PCR reaction tube (Pang et al., 2020). After 30 min of RT-LAMP amplification, the amplification product is mixed with the CRISPR-Cas12a detection reagent, generating fluorescence immediately and detecting within 10 min. Clinical testing showed that the method had 100% clinical specificity and 94% clinical sensitivity. Overall, the “one pot” test simplifies the operation and minimizes the risk of cross-contamination.

### 5.4 Perspectives and chanllenges of CRISPR-cas biosensor technology on POCT

CRISPR-Cas technology is the next-generation of biosensor diagnostic platform for nucleic acid detection. It has two categories for on-site detection: DNA (HOLMES, DETECTR, Cas12aVDet) (Chen et al., 2018; Wang et al., 2019) and RNA (HUDSON, SHERLOCK, etc.) (Kellner et al., 2019). To improve on-site detection, some key problems need to be solved such as developing a new type of Cas protein to target specific genes and expanding the target detection range (Liu et al., 2021b; Fozouni et al., 2021). Isothermal amplification can achieve ultra-high sensitivity detection, but it is vulnerable to aerosol pollution resulting in false positive interpretation. One tube method and lateral flow test strips or portable fluorescence detection can reduce the occurrence of false positive, but can only achieve qualitative nucleic acid detection (Barnes et al., 2020). Achieving quantitative nucleic acid detection while meeting the requirements of portable detection is a direction for future research. The effect of on-site clinical testing and reducing testing cost are areas that require more R&D investment. CRISPR-Cas technology is suitable for on-site nucleic acid detection and has potential for wider application in the future.

The use of CRISPR-Cas in nucleic acid POCT faces many challenges, including limited detection due to sequence restrictions, the need for careful selection of effector proteins and sensing regions, and the standardization of results interpretation and operating procedures. Quantitative analysis is also important in nucleic acid detection, but most current CRISPR-Cas methods only provide qualitative data. To address these challenges, CRISPR technology is being integrated into various traditional and new detection methods, such as electrochemical detection, transverse flow analysis, nano material sensors, microfluidics, and hydrogels.

The CRISPR-Cas detection technology is promising, but still in its early stages. As genomics develops, more CRISPR systems are being discovered, offering opportunities to explore unknown functions and revolutionize nucleic acid POCT analysis. Combining different strategies from materials science and cross science will help solve existing problems in CRISPR-Cas application and make significant contributions to rapid nucleic acid detection.

## 6 Applications of CRISPR-cas system for SARS-CoV-2 diagnostic test

SARS-CoV-2 has become a global health crisis infecting over 120 million people and causing almost 2 million deaths in more than 200 countries (Sanche et al., 2020). Infected individuals can spread the virus before or without symptoms, making rapid and sensitive diagnostic testing crucial. RT-qPCR is currently the preferred diagnostic method, but requires trained technicians and is slowed by a lack of reagents and equipment. CRISPR-based detection methods, particularly those using Cas12 and Cas13, offer accurate and rapid detection and are a promising new approach for treating and detecting SARS-CoV-2. SARS-CoV-2 invasion is primarily mediated by human angiotensin-converting enzyme 2 (hACE2). Recent developments in ACE2-based SARS-CoV-2 detection modalities accentuate the potential of this natural host-virus interaction for developing point-of-care (POC) COVID-19 diagnostic systems (Gul et al., 2022b).

CRISPR-Cas system has been studied for its potential in screening therapeutic targets for SARS-CoV-2 (Abbott et al., 2020; Hoffmann et al., 2021; Wei et al., 2021). Several studies have used CRISPR-Cas to target proteins interacting with SARS-CoV-2, screen host factors needed for coronavirus infection, and establish whole genome screening systems (Abbott et al., 2020; Hoffmann et al., 2021; Wei et al., 2021). In addition, a viral sequence degradation therapy based on CRISPR-Cas13 system has been developed to effectively degrade SARS-CoV-2 RNA sequence and influenza A virus RNA sequence in human lung epithelial cells, potentially becoming an effective strategy to suppress SARS-CoV-2 (Nguyen et al., 2020). Furthermore, the use of adenovirus vector to import CRISPR-Cas13d system and multiple guide RNAs targeting the polypeptide coding region of SARS-CoV-2 virus into COVID-19 patients has shown promise in limiting virus replication and inhibiting the harm of virus to human body, providing a new clinical treatment method for the treatment of mutant RNA viruses that have developed drug resistance (Nguyen et al., 2020).

The novel coronavirus (SARS-CoV-2) has a genome structure containing 29,903 nucleotides, including genes encoding unstructured, spike, envelope, membrane, and nuclear proteins (Chan et al., 2020a; Chan et al., 2020b; Lu et al., 2020). Research shows that 8 types of coronaviruses can cause respiratory diseases, including 3 α Coronaviruses (HCoV-229E, HKU-NL63, CCoV HuPn-201) and 5 β Coronaviruses (HCoV-OC43, HCoV-HKU1, SARS CoV, MERS CoV, and SARS-CoV-2) (Vlasova et al., 2022). SARS-CoV-2 is a highly transmissible member of βcoronavirus, causing SARS-CoV-2 pneumonia and posing significant threats to human life and health.

Currently, there are various methods for detecting SARS-CoV-2, such as gene sequencing, nucleic acid molecular diagnosis, immunological diagnosis, and imaging diagnosis. Each method has its pros and cons as listed in Table 3 (Ji et al., 2020; Kruttgen et al., 2021). RT-qPCR is the most reliable method for diagnosing SARS-CoV-2, but it requires expensive equipment and skilled operators, making it difficult to perform real-time detection at all times. Moreover, many RT-qPCR kits on the market have inconsistent quality, resulting in low sensitivity and specificity, which can lead to false negative results and misdiagnosis (Taleghani and Taghipour, 2021).

**TABLE 3:** Comparisons of advantages and disadvantages of various SARS-Cov-2 diagnosis methods.

Characteristics	Detection methods					
	Gene sequencing	Gene chip	CLIA	RT-LAMP	CRISPR-cas	RT-qpcr
Advantages	High accuracy	High degree of automation, high efficiency and low cost	High sensitivity, high specificity, fast speed	High sensitivity, simple operation, low equipment requirements	High specificity	High specificity and accurate results
Disadvantages	Time-consuming and costly	High requirements for equipment and operators	High cost, high requirements for equipment and operators	High false positive rate	Low sensitivity	High false negative rate, high requirements for equipment

### 6.1 Subtype of CRISPR-cas system used for SARS-CoV-2 detection

Advancements in technology, including third-generation sequencing, lower genome sequencing costs, and comparative analysis of human genome sequences, have increased the accuracy of genetic disease diagnosis. CRISPR-Cas technology has broad applications in gene editing, disease treatment, model organism construction, and plant breeding. The isothermal nucleic acid amplification technology of the CRISPR-Cas system has played a significant role in genetic diagnosis. Below, we will introduce various CRISPR-Cas systems and their use in detecting SARS-COV-2.

#### 6.1.1 CRISPR-Cas9 for SARS-CoV-2 detection

Xiong et al. developed a rapid dual gene detection method for SARS-CoV-2 using the CRISPR-Cas9 system and RT-RPA with a sensitivity of 4 cps/μl. Marcic et al. developed the low-cost Vigilant detection technology with ultra-high sensitivity and no cross reaction with SARS CoV or MERS CoV (Xiong et al., 2021). Moon et al. reported a virus colorimetric detection method based on CRISPR-dCas9 that successfully recognized SARS-CoV-2, pH1N1, and pH1N1/H275Y with the naked eye (Moon et al., 2020). Jiao et al. developed the LEOPARD diagnostic platform, which can simultaneously detect and distinguish SARS-CoV-2 and its D614G variant in patient samples (Jiao et al., 2021). Currently, Cas9 protein is widely used as a tool for gene knockout in basic research but has not been used in clinical diagnosis.

#### 6.1.2 CRISPR-Cas12 for SARS-CoV-2 detection

Cas12a protein is an endonuclease belonging to type V protein in CRISPR-Cas system of class II that recognizes target sequences to activate non-specific cleavage of single strand DNA (ssDNA). SARS-CoV-2 DETECTR detection system is developed using Cas12 protein as it can cleave ssDNA non-specifically by adding a fluorescent molecule connected to the quenching agent through ssDNA, resulting in a change of colour observed visually to achieve qualitative and quantitative detection (Broughton et al., 2020). Other detection platforms based on CRISPR-Cas12 system are slightly less sensitive than reverse transcription polymerase chain reaction (RT-PCR). A new crRNA was designed to reduce the loss of the target DNA sequence. The detection system can realize isothermal amplification and Cas12 protein cleavage in a reaction tube, thus reducing pollution, improving sensitivity and realizing one-step visual detection of SARS-CoV-2 (Wang et al., 2021). Reverse transcription is required when detecting some RNA samples, and the steps are a little cumbersome.

**FIGURE 3:**
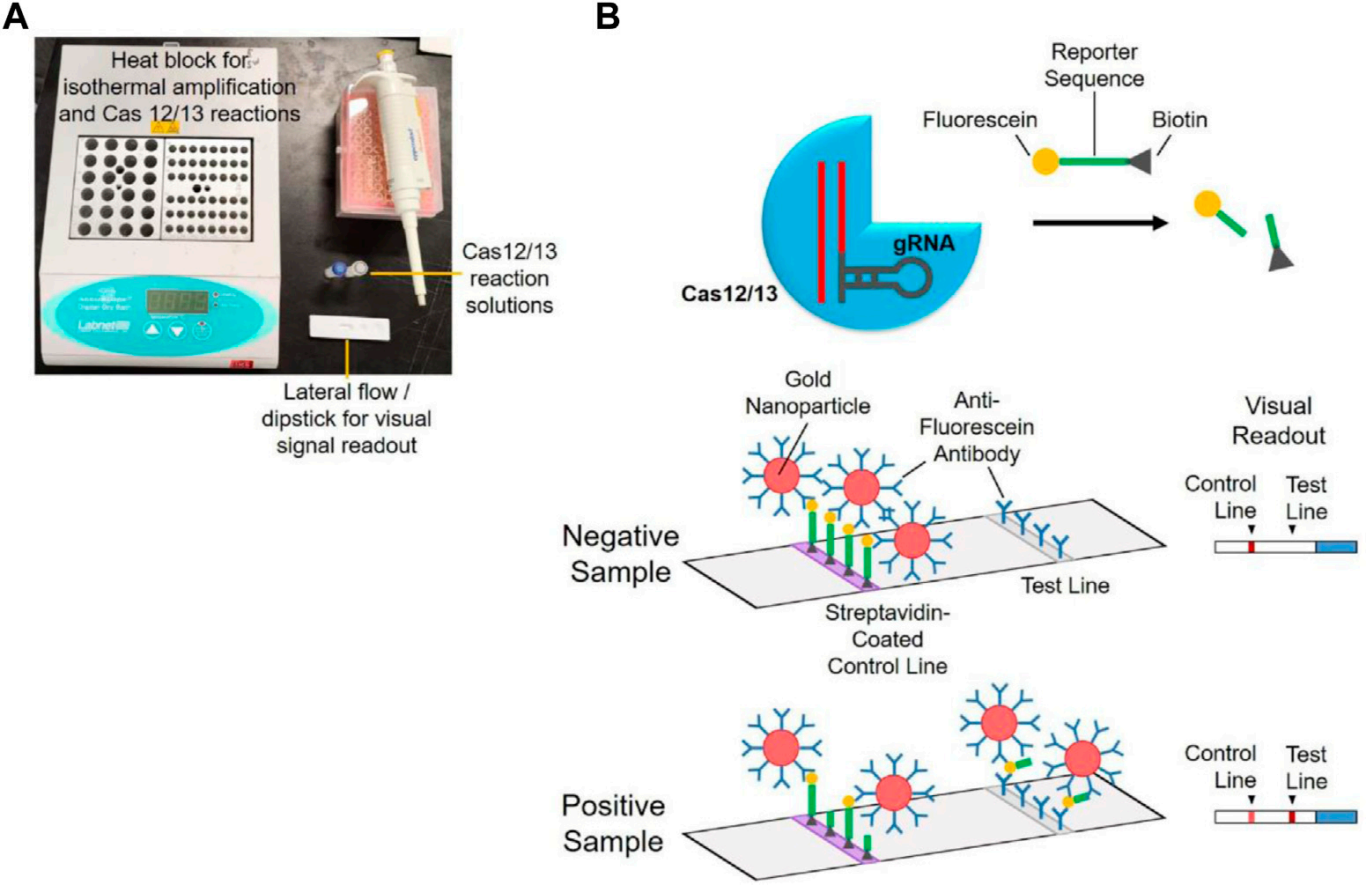
Diagram for the basic steps of using CRISPR-Cas12/13 assay for SARS-CoV-2 detection. **(A)** Simple instrumentation needed for POC testing. **(B)** Mechanism for visual signal readout using a lateral flow dipstick. Reprinted with permission from Soh et al. (2022). Copyright ^©^ 2022 Elsevier Ltd Terms and Conditions.

Researchers have developed several new diagnostic methods based on the CRISPR system to detect SARS-CoV-2. Using the detection platform of the CRISPR-Cas12a system, viral RNA from nasal swabs can be amplified and transferred for fluorescence detection, providing rapid results (Broughton et al., 2020). A lateral flow assay can also be used for detection in respiratory tract extracts. Additionally, a rapid, naked-eye visual detection method (CRISPR-Cas12a-NER) has been developed, providing a simple and reliable field diagnosis method (Wang et al., 2020f). A combination of isothermal amplification and CRISPR-Cas12 technology has been used for clinical specimens, greatly shortening detection time and providing accurate results (Xiang et al., 2020). See Figure 3 for the basic steps of using the CRISPR-Cas12/13 assay for SARS-CoV-2 detection.

#### 6.1.3 CRISPR-Cas13 for SARS-CoV-2 detection

Cas13a protein is a component of the CRISPR-Cas system of type 1, specifically belonging to type VI. It forms a ribonucleoprotein complex (RNP) with crRNA that does not activate nuclease. When RNP and complementary target RNA combine, the HEPN motif of Cas13 protein is activated, allowing for non-specific cutting of ssRNA. This characteristic can be used to directly detect RNA samples. The SHERLOCK detection system, developed in 2017 by Joung et al. (Joung et al., 2020a), combines isothermal nucleic acid amplification with Cas13a protein cutting for high sensitivity and specificity in detecting SARS-CoV-2. The STOPCovid.V2 detection method, developed later by Joung et al., combines the CRISPR-Cas13 system with LAMP technology for one-step direct detection of RNA viruses (Joung et al., 2020b). Fozouni et al. have also utilized multiple crRNAs with different specific sequences to accelerate Cas13a protease activation for accurate detection of SARS-CoV-2 (Fozouni et al., 2021). The development of Cas13 protein has greatly improved the efficiency of RNA virus detection and is expected to further improve the clinical detection of SARS-CoV-2.

The SHERLOCK technology based on CRISPR is being used as a new method to detect SARS-CoV-2 during the COVID-19 outbreak. It consists of three steps that take less than an hour from nucleic acid extraction, and is used for quantitative reverse transcription polymerase chain reaction detection. The method involves isothermal amplification, detection using Cas13, and reading out the results with the aid of instruments. The SHINE method, developed by Arizti-Sanz and team, can be used with SHERLOCK to detect SARS-CoV-2 RNA in unextracted samples (Arizti-Sanz et al., 2020). It can quickly and sensitively detect viral RNA in patient samples with minimal equipment, reducing the risk of contamination. This method has been highly sought after during the pandemic, and has improved diagnostic testing capabilities for infectious diseases.

#### 6.1.4 CRISPR-Cas14 for SARS-CoV-2 detection

Cas14 protein is the smallest class II CRISPR effector protein found to date that can perform DNA cleavage. It is composed of 400–700 amino acids and has non-specific incidental cleavage activity like Cas12 and Cas13 proteins. However, Cas14a protein has stronger specific recognition ability for ssDNA and is not limited by PAM sequence, making it useful in nucleic acid detection systems for single base mutation. Harrington et al. improved the Cas14 effector protein on the basis of DETECTR, resulting in the Cas14-DETECTR system which can quickly detect infectious organisms and gene mutations using Cas12 and Cas13 proteins (Harrington et al., 2018). Although no research has shown that Cas14 protein can be used for the detection of SARS-CoV-2, its small size and lack of limitation by PAM sequence make it a potential tool protein for gene therapy.

### 6.2 Comparision of various SARS-CoV-2 test methods based on CRISPR-cas and RT-qPCR

Clinical studies found that patients with SARS-CoV-2 recovered after treatment, but traditional RT qPCR detection methods may not be sensitive enough. More sensitive detection methods, such as the ultra-sensitive nucleic acid detection method based on CRISPR Cas system, are needed to detect the viral load in patients. This method has advantages such as simple operation, requiring less instruments and equipment, high sensitivity and specificity (Table 4) and is expected to replace RT-qPCR for SARS-CoV-2 detection in the future.

**TABLE 4:** Comparisons of various SARS-CoV-2 test methods based on CRISPR-Cas and RT-qPCR.

Detection method	Type of cas protein used	Target genes detected	Sensitivity (copy number/μl)	Specificity	Detection steps	Detection time (min)
RT-qPCR	Not Applicable	ORFlab, N	500	High	1	120
SHERLOCK	Cas13	ORFlab, S	10–100	High	3	60
STOPCovid.V2	Cas12b	N	100	High	1	15–45
AIOD-CRISPR	Cas12a	N	1.3	High	1	40
DETECTR	Cas12a	N, E	10	High	2	30
Opv-CRISPR	Cas12a	S	5	High	1	5

### 6.3 Advantages of SARS-CoV-2 nucleic acid detection based on crispr technology: High efficiency, accuracy and visualization

Clinical studies show that hospitalized patients’ virus titer fluctuates day-to-day and has no relation to the severity of the disease (Qu et al., 2020). For 24 recovered patients, commercial kits detected negative RNA for both the N and ORF1b genes a few days after readmission, while the SHERLOCK system had a higher sensitivity of 0.1 Mcopies/L. 75% of samples were positive for the S gene and 41.6% for the ORF gene during convalescence (Patchsung et al., 2020). Therefore, more sensitive nucleic acid detection methods are needed. Hou et al. established a CRISPR-nCoV method that had close to single-copy sensitivity and shorter detection time than RT-PCR (Hou et al., 2020). Broughton et al. developed a side-flow detection method for SARS-CoV-2 using a loop-mediated isothermal amplification reaction and CAS12 detection, with a detection limit of 10 Mcopies/L on a side-flow tomography strip and a detection time of only 30min (Broughton et al., 2020). Zhang et al.‘s SHERLOCK SARS-CoV-2 nucleic acid detection technology based on CRISPR can detect the target sequence in the range of 20–200 a.m. within 60min by using artificially synthesized RNA fragment of SARS-CoV-2 virus (Gootenberg et al., 2017). Thus, an ultra-sensitive method based on CRISPR is expected to be used for accurate and effective monitoring and management of SARS-CoV-2 patients during convalescence.

Furthermore, CRISPR-nCoV is a promising new method for detecting SARS-CoV-2 with high sensitivity and specificity (Hou et al., 2020). Broughton et al.‘s lateral flow chromatographic detection method based on CRISPR (SARS-CoV-2 DETECTR) provides a rapid and visual detection method for SARS-CoV-2 (Joung et al., 2020a). Meanwhile, Patchsung et al. showed that the specificity and sensitivity of SHERLOCK method in detecting SARS-CoV-2 were 100% and 97% (Patchsung et al., 2020). Therefore, the CRISPR-based method is expected to be used for specific detection of SARS-CoV-2, which is crucial for epidemic prevention and control.

Efficient and accurate SARS-CoV-2 nucleic acid detection is critical for controlling the global spread of the virus. Current methods require specimens to be transported to a designated lab, which takes over 24 h, and are performed by well-trained personnel with expensive equipment. This is not conducive to effective monitoring of the epidemic. Isothermal amplification methods are a simple, rapid, and low-cost alternative to traditional PCR methods, but developing them into reliable point-of-care testing (POCT) for clinical application remains a challenge. Researchers have developed isothermal amplification methods based on CRISPR technology that can overcome these challenges. These methods boast high detection sensitivity and rapid detection of target sequences, and are expected to be developed into portable visual detection kits. Examples of such methods include the AIOD-CRISPR, StopCoVid, and CRISPR-Cas12 methods, which have the potential to revolutionize molecular diagnosis of SARS-CoV-2.

Quick and accurate identification of infectious diseases is crucial for effective clinical management, guiding infection control and public health interventions. Nucleic acid detection methods, such as RT-PCR and Metagenomic next-generation sequencing (mNGS), have different advantages and limitations (Li, 2022). Although IgG/IgM detection kits have a high false positive rate and are not suitable for clinical use alone, they can be used as a complementary option for RT-PCR. The CRISPR-based method is faster than RT-PCR and mNGS, taking only 40 min to complete the entire detection process. It has advantages such as isothermal amplification and easy combination with other methods, making it suitable for development into a field detection kit. The CRISPR-based detection method is expected to be developed into a clinical detection kit for the rapid and sensitive detection of SARS-CoV-2 in medically underdeveloped and remote areas.

## 7 Conclusion

CRISPR-Cas is a cutting-edge technology that can be used to develop antiviral treatments and molecular diagnostics that are specific, rapid, and easy to use even in low-resource settings. This technology can be used to diagnose SARS-CoV-2 with high accuracy and sensitivity, and can detect the virus in various types of specimens. Compared to existing methods, CRISPR-based methods have several advantages, including portability, low cost, and rapidity, making them ideal for use in epidemic areas and remote regions with limited medical resources.
